# Peripheral myeloid cells contribute to brain injury in male neonatal mice

**DOI:** 10.1186/s12974-018-1344-9

**Published:** 2018-10-30

**Authors:** Peter L. P. Smith, Amin Mottahedin, Pernilla Svedin, Carl-Johan Mohn, Henrik Hagberg, Joakim Ek, Carina Mallard

**Affiliations:** 1 Institute of Neuroscience and Physiology, Department of Physiology, Sahlgrenska Academy, University of Gothenburg, Box 432, SE-405 30 Gothenburg, Sweden; 2 Institute of Clinical Sciences, Department of Obstetrics and Gynaecology, Sahlgrenska Academy, University of Gothenburg, Gothenburg, Sweden

**Keywords:** Neuroinflammation, Newborn, Immune cell trafficking

## Abstract

**Background:**

Neonatal brain injury is increasingly understood to be linked to inflammatory processes that involve specialised CNS and peripheral immune interactions. However, the role of peripheral myeloid cells in neonatal hypoxic-ischemic (HI) brain injury remains to be fully investigated.

**Methods:**

We employed the *Lys-*EGFP-*ki* mouse that allows enhanced green fluorescent protein (EGFP)-positive mature myeloid cells of peripheral origin to be easily identified in the CNS. Using both flow cytometry and confocal microscopy, we investigated the accumulation of total EGFP^+^ myeloid cells and myeloid cell subtypes: inflammatory monocytes, resident monocytes and granulocytes, in the CNS for several weeks following induction of cerebral HI in postnatal day 9 mice. We used antibody treatment to curb brain infiltration of myeloid cells and subsequently evaluated HI-induced brain injury.

**Results:**

We demonstrate a temporally biphasic pattern of inflammatory monocyte and granulocyte infiltration, characterised by peak infiltration at 1 day and 7 days after hypoxia-ischemia. This occurs against a backdrop of continuous low-level resident monocyte infiltration. Antibody-mediated depletion of circulating myeloid cells reduced immune cell accumulation in the brain and reduced neuronal loss in male but not female mice.

**Conclusion:**

This study offers new insight into sex-dependent central-peripheral immune communication following neonatal brain injury and merits renewed interest in the roles of granulocytes and monocytes in lesion development.

**Electronic supplementary material:**

The online version of this article (10.1186/s12974-018-1344-9) contains supplementary material, which is available to authorized users.

## Background

Inflammation is widely recognised as an important component of perinatal brain injury [1, 2]. Persistent inflammation is thought to negatively impact ongoing developmental processes and potentially sensitise to later life pathologies [3, 4]. The central nervous system (CNS), with its developmentally distinct population of mononuclear phagocytes [5], immune-suppressive environment and highly regulated interactions with the innate and adaptive arms of the immune system [6], is viewed as an immune-specialised organ [7]. In response to pathological insult, the immature CNS actively upregulates numerous chemoattractant molecules including the binding partners of CCR2 (CCL2 and CCL7), CCR1 and CCR5 (CCL3) and CXCR2 (CXCL1) [8], respectively known for their roles in emigration of Ly6C^hi^ monocytes from the bone marrow and recruitment of monocytes into inflamed tissue [9]. Indeed, accumulation of macrophages [10], neutrophils [11–13], mast cells [14] and NK cells [11] occurs in response to neonatal hypoxia-ischemia (HI). An important question yet to be satisfactorily addressed in neonatal injury models is the relative contribution of microglial-derived macrophages (MiDMs) vs that of monocyte-derived macrophages (MDMs) to the CNS macrophage pool: historically, discrimination between these cell types has proved difficult due to their assumed similar morphology and common expression of cell surface epitopes such as Fc and complement receptors, CD11b, F4/80 [15] and CD45 [16]. Despite such shared characteristics, MiDMs and MDMs are increasingly appreciated to play differing roles in the context of CNS insult [17]. Matters are further complicated by the non-homogenous nature of blood-borne monocytes; at least two distinct subsets have been identified and classified as inflammatory and resident monocytes [18]. Inflammatory monocytes represent a relatively short-lived population that is actively recruited to inflamed tissue [18, 19], whereas resident monocytes are physiologically recruited to non-inflamed tissue [18] and have the capacity to rapidly respond to tissue damage or infection [20]. These monocyte subsets display differential migratory dynamics in adult cerebral ischemia: inflammatory monocytes make a rapid but transient appearance, while resident monocytes display a delayed but progressive accumulation [21]. The inflammatory characteristics of each subset may underpin such dynamics: inflammatory monocytes upregulate inflammatory mediators including *TNFα* and *IL1*, while resident monocytes display a more reparative phenotype with elevated expression of genes involved in tissue remodelling such as *arg1* and *Fizz1* [20], drawing comparisons respective to M1 and M2 macrophage phenotypes [22].

Here, we employed immunohistochemistry and flow cytometry to investigate MDM and granulocyte infiltration in the post-ischemic neonatal brain. We performed experimental HI on postnatal day (P) 9 *Lys*-EGFP-*ki* mice, allowing identification of peripheral myeloid cells in the brain [23, 24]. For the first time, we describe the differential dynamics of resident and inflammatory monocytes in this model and that inhibition of myeloid cell accumulation in the brain protects against HI injury in male, but not female, neonatal mice.

## Methods

### Animals

Pregnant C57BL/6J dams were sourced from Janvier Laboratories (Le Genest-Saint-Isle, Fr). *Lys-*EGFP-*ki* mice were obtained from Dr. Tomas Graf, Autonomous University of Barcelona [22]. Animals were housed and bred at the University of Gothenburg’s Laboratory for Experimental Biomedicine on a 12-h light-dark cycle (illuminated 07:00–19:00) at constant temperature (24 °C) and relative humidity (50–60%) with ad libitum access to food and water. All experimental procedures were approved by the Gothenburg Animal Research Ethics Committee (No. 337/2012, 139/2013, 18/2015).

### Experimental hypoxia-ischemia

HI brain injury was induced in male and female mice on postnatal day (P) 9. Pups with body weight < 4 g at the time of HI were excluded from experiments. The mortality rate was < 5% throughout the study. A total of 306 animals were included in the study. Briefly, mice were anaesthetised with isoflurane in a 1:1 nitrous oxide to oxygen mix (4% induction, 2% maintenance) and subjected to permanent occlusion of the left common carotid artery. Mice were then allowed a 1-h recovery period before being transferred to a temperature-controlled (36 °C) humidified incubator for 50 min of hypoxia (10% O_2_). Sham animals were subjected to anaesthesia, and the carotid artery was exposed as above but without ligation of the artery and hypoxia.

### EGFP, CD31, IBA1 and Ly6G immunohistochemistry

Mice were deeply anaesthetised and transcardially perfused with ice-cold 0.9% saline followed by 4% paraformaldehyde (PFA). Brains were rapidly removed, post-fixed in 4% PFA for 24 h at 4 °C and cryoprotected in 30% sucrose for a minimum of 3 days. Cryoprotected brains were snap-frozen on dry ice and sectioned serially at 40 μm on a Leica CM3050S cryostat (Leica, SE). Cut sections were transferred to a cryoprotectant solution (25% ethylene glycol, 25% glycerine, in 0.1 M phosphate buffer) and stored at − 20 °C. Sodium citrate antigen retrieval (10 mM sodium citrate, pH 6, 97 °C, 10 min) was performed prior to all staining procedures. Blocking of non-specific binding sites was achieved through a 30-min incubation in Tris-buffered saline (TBS) containing 3% donkey serum (hereafter referred to as blocking buffer). Sections were then incubated at 4 °C overnight with given combinations of primary antibodies which were later visualised via a 2-h room temperature incubation with relevant secondary antibodies (see Table 1).

**Table 1:** Antibodies for immunohistochemistry and flow cytometry

Application	Antigen	Host	Clone/target	Reactivity*	Conjugate	Company	Product number	Dilution**
Immunohistochemistry	GFP	Rabbit	Poly	Mouse	A488	Invitrogen	A21311	1:200
	CD31	Goat	Poly	Mouse	–	R&D systems	AF3628	1:200
	Iba1	Goat	Poly	Mouse	–	Abcam	Ab5076	1:500
	Ly6G	Rat	Mono (1A8)	Mouse	–	BioLegend	127602	1:250
	IgG	Donkey	Poly	Goat	CF555	VWR	89138-464	1:1000
	IgG	Goat	Poly	Rat	CF555	Sigma	SAB46000070	1:1000
Flow cytometry	CD11b	Rat	M1/70	Mouse	PE-Cy7	BioLegend	101216	0.25 μg
	CD45	Rat	30-F11	Mouse	APC-CY7	BD	557659	0.25 μg
	GR-1	Rat	RB6-8C5	Mouse	PerCp-Cy5.5	eBiosciences	45-5931	0.25 μg
	Ly6C	Rat	HK1.4	Mouse	APC	eBiosciences	17-5932	0.25 μg
	CD16/CD32	Rat	2.4G2	Mouse	–	BD	553142	0.1 μg

*Where antibody is reactive to multiple species, only relevant species are listed

**For flow cytometry, given values represent microgram antibody per 10^6^ cells

### Microscopy

Tile-scanned images of entire brain sections were captured on a Zeiss Axio Observer upright microscope equipped with an Apotome module and Zen blue software (Zeiss, Oberkochen, DE). From each experimental group (*n* = 4), 2–3 sections at hippocampus and striatum levels were analysed. Each *z*-stack consisted of 8–10 images with a *z*-plane distance of 3 μm. All other images were captured using a Zeiss LSM 700 inverted confocal (Zeiss, Oberkochen, DE). *Z*-projections were produced in ImageJ (NIH, Bethesda, http://rsbweb.nih.gov/ij/), and figures were compiled in Adobe CS6.

### Tissue collection and preparation for flow cytometry

Brain samples were collected at 6 h, 1 day, 3 days, 7 days, 14 days and 28 days after HI. Mice were deeply anaesthetised and transcardially perfused with ice-cold 0.9% saline, brains were rapidly removed and dissected hemispheres were transferred to ice-cold Hanks’ Balanced Salt Solution (HBSS) containing 0.5% bovine albumin serum (BSA) (hereafter referred to as FACS buffer) and kept on ice until dissociation. Single-cell suspensions were obtained through enzymatic dissociation (0.01% papain [Bionordika, Stockholm, SE], 0.1% dispase II [Bionordika, Stockholm, SE], 0.01% DNase I [Roche, Bromma, SE], 12.4 mM MgSO_4_ in Ca/Mg-free HBSS); briefly, samples underwent three rounds of enzymatic (10-min incubation at 37 °C) and mechanical (repetitive pipetting) dissociation. Resultant samples were passed over a 40-μm cell strainer, centrifuged (500*g*, 5 min), resuspended in FACS buffer and quantified on a BioRad TC10 automated cell counter (BioRad, Solna, SE). Samples were incubated for 15 min at 4 °C with Fc block (CD16/CD32) and then primary antibodies in relevant combinations (Table 1). Stained samples were centrifuged (500*g*, 5 min), re-suspended in FACS buffer and kept at 4 °C until analysis.

### Flow cytometry

**Fig. 1:**
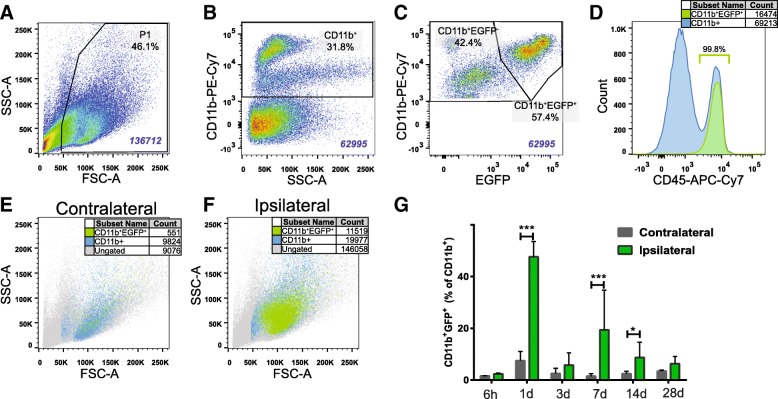
EGFP^+^ myeloid cells in the brain after hypoxia-ischemia. **a–c** Gating strategy applied to all samples displaying cells isolated from the ipsilateral hemisphere at 24 h after hypoxia-ischemia (HI). Single-cell suspensions derived from ipsilateral and contralateral hemispheres of *Lys*-EGFP-*ki* mice were gated based on size (forward scatter) and granularity (side scatter) (**a**) followed by CD11b immunoreactivity (**b**) and EGFP expression (**c**). **d** EGFP^+^ cells display CD45^hi^ expression; *n* = 12. **e**, **f** Backgating shows CD11b^+^EGFP^+^ in the contralateral (**e**) and ipsilateral (**f**) hemispheres 24 h after HI. **g** Compiled data displaying presence of CD11b^+^EGFP^+^ infiltrating cells at 6 h (*n* = 8), 1 day (*n* = 7), 3 days (*n* = 23), 7 days (*n* = 19), 14 days (*n* = 14) and 28 days (*n* = 9) after HI. Values are presented as the mean ± SD. One-way ANOVA followed by Holm-Sidak’s post hoc test comparing differences between hemispheres at each time point. *p ≤ 0.05, ***p ≤ 0.001

Cell viability was determined, in pilot experiments using 7AAD, to be 98.47 ± 0.39% (*n* = 12). Debris were excluded by gating on size and granularity (P1 gate; Fig. 1a). Myeloid cells were identified by CD11b expression; a mean (from all analyses presented herein) of 38,000 CD11b^+^ cells per sample was analysed (Figs. 1a–c and 4a for gating strategies). CD11b^+^EGFP^+^Ly6C^+^ cells were considered myeloid cells of peripheral origin and further categorised by differential Ly6C expression as inflammatory monocytes (CD11b^+^EGFP^+^Gr1^lo/−^Ly6C^int/hi^), resident monocytes (CD11b^+^EGFP^+^Gr1^lo/−^Ly6C^lo/−^) [25] or granulocytes (CD11b^+^EGFP^+^Gr1^hi^Ly6C^int^) [26]. All data was collected using a BD FACSCanto flow cytometer with BD FACSDiva software v.6.1.3 (BD Biosciences, Stockholm, SE); analysis was performed with FlowJo v.10 (Tree Star Inc., Ashland, OR, USA).

### Bio-plex cytokine analysis

Brain and plasma samples were collected at 6 h, 1 day, 3 days, 7 days and 14 days after HI. Briefly, mice were deeply anaesthetised, blood was collected from the heart’s right ventricle and transferred to EDTA-coated tubes and set aside for further processing, animals were then transcardially perfused with ice-cold 0.9% saline and brains were rapidly removed and frozen on dry ice. Plasma samples were isolated via centrifugation (10 min, 1000×*g*, 4 °C) and frozen on dry ice prior to storage at − 80 °C.

Brain lysates were prepared through mechanical dissociation of tissue samples in 600 μl of lysis buffer (10 mM EDTA, 1% Triton-X-100, 1% Protein Inhibitor Cocktail [Sigma-Aldrich#8340] in RNase-free PBS) followed by sonication and centrifugation (4500×*g*, 5 min, 4 °C). Protein concentration was assessed using a Pierce BCA Protein Assay Kit (Thermo Fisher Scientific) as per manufacturer’s protocol, and the final concentration of the samples was adjusted to 1 mg/ml using the lysis buffer. Preparations were carried out at 4 °C, and samples were stored at − 80 °C.

Cytokine concentration in brain lysates and plasma samples were assessed using a Bio-Plex Pro Mouse Cytokine Standard 23-Plex (Bio-Rad) kit in accordance with manufacturers’ instructions on a Bio-Plex 200 analyser. For brain samples, the results were normalised to the brain protein concentration.

### Antibody-based depletion of circulating monocytes and neutrophils

Circulating monocytes and neutrophils were depleted via intraperitoneal (i.p.) administration of GR-1 antibody (clone RB6-8C5, 17 mg/kg; Bio X Cell# BE0075). Administration commenced 1 h after removal of mice from the hypoxia chamber and was repeated every 48 h thereafter until sacrifice. The antibody dose was selected based on previous work with a similar concentration that demonstrated successful depletion of monocytes and neutrophils in 8–12-week-old mice [27].

### Brain injury analysis

Mice exposed to HI and treated with saline or RB6-8C5 antibody for 2 weeks, as described above, were deeply anaesthetised on P23 (i.e. 14 days after HI for assessing long-term outcome) and transcardially perfused with ice-cold 0.9% saline followed by 4% PFA. Brains were rapidly removed, post-fixed in 4% PFA for 24 h at 4 °C, dehydrated and embedded in paraffin. Brains were then serially cut at 10 μm (at 50-section intervals) on a Leica RM2165 microtome (Leica, SE). Three sections spanning the hippocampus (Fig. 7a) of each brain were analysed. Antigen retrieval was performed through boiling sections in sodium citrate buffer for 10 min, and non-specific binding was blocked by a 30-min incubation in PBS containing 1% horse serum, 3% BSA and 0.1% NaN_3_. Sections were then incubated with antibodies against microtubule-associated protein-2 (MAP2) (clone HM.2, 1:1000; Sigma-Aldrich) or myelin basic protein (MBP) (clone SMI-94R, 1:10,000; Covance) overnight at 4 °C. Biotinylated secondary antibodies were applied for 1 h at room temperature and visualised using a Vectastatin ABC Elite followed by standard DAB staining. Brain images were captured using a Nikon Optiphot-2 microscope equipped with AVT dolphin F145B camera (Allied Vision Technologies). Percent loss of MAP2-positive tissue was calculated by subtracting the MAP2-positive area in the ipsilateral hemisphere from that measured in the contralateral hemisphere, then dividing the result by the MAP2-positive area of the contralateral hemisphere and converting to percent tissue loss. Percent loss of MBP-positive tissue was calculated in the same manner.

### Statistics

Data are presented as group mean ± standard deviation (SD). Comparisons between ipsilateral and contralateral changes in number of infiltrating cells over time were assessed by one-way ANOVA followed by Holm-Sidak’s multiple comparison tests. Differences were considered significant at *p < 0.05, **p < 0.01 and ***p < 0.001. Brain injury comparisons were performed using multiple unpaired Student’s *t* tests at each brain level; *p* values were corrected for multiple comparisons using the Holm-Sidak method. Differences were considered significant at *p < 0.05. Analyses were performed using Prism (Graphpad, v.6.05).

## Results

### Peripheral immune cells are detected in the CNS for up to 14 days after experimental HI

To assess the potential influx of peripheral immune cells to the CNS following HI brain injury, we subjected P9 *lys*-EGFP-*ki* mice to experimental HI, collected tissue at 6 h, 1 day, 3 days, 7 days, 14 days and 28 days after HI and employed flow cytometry to quantitatively assess the presence of EGFP^+^ infiltrating cells in injured vs uninjured cerebral hemispheres. Infiltrating myeloid cells were identified through a stepwise gating strategy: cells were first gated by size and granularity (Fig. 1a), followed by CD11b (Fig. 1b) and finally EGFP expression (Fig. 1c). We found that 99.80% ± 0.06% of cells identified as CD11b^+^EGFP^+^ were CD45^hi^, confirming their peripheral origin (Fig. 1d). CD11b^+^EGFP^+^ infiltrating myeloid cells were significantly increased in the ipsilateral compared with the contralateral hemisphere at 1 day (*p* < 0.001), 7 days (*p* < 0.001) and 14 days (*p* = 0.031) after HI (Fig. 1e–g), with CD11b^+^EGFP^+^ cells respectively constituting 47.65 ± 2.40%, 19.41 ± 3.51% and 8.75 ± 1.56% of the injured hemisphere’s total CD11b^+^ cell population (Fig. 1g).

**Fig. 2:**
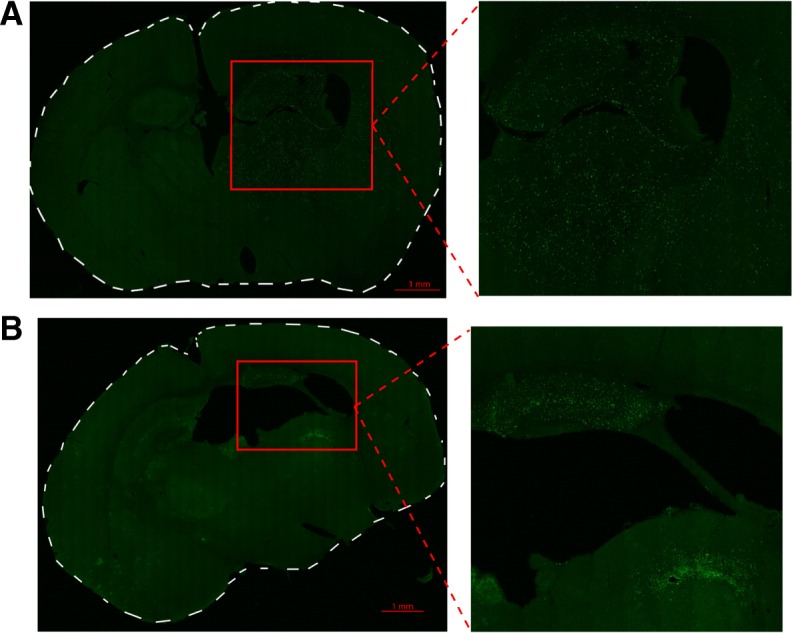
EGFP^+^ myeloid cell localisation in the brain after hypoxia-ischemia. **a** Representative tile-scanned confocal images of brain sections after hypoxia-ischemia (HI). **a** Dispersed pattern of EGFP^+^ myeloid cell infiltration in the hippocampus and thalamus 1 day after HI. **b** EGFP^+^ myeloid cells localised in the hippocampus and the white matter of the thalamus (medullary lamina of thalamus) in a spatially limited dense pattern 7 days after HI. *n* = 4/time point

**Fig. 3:**
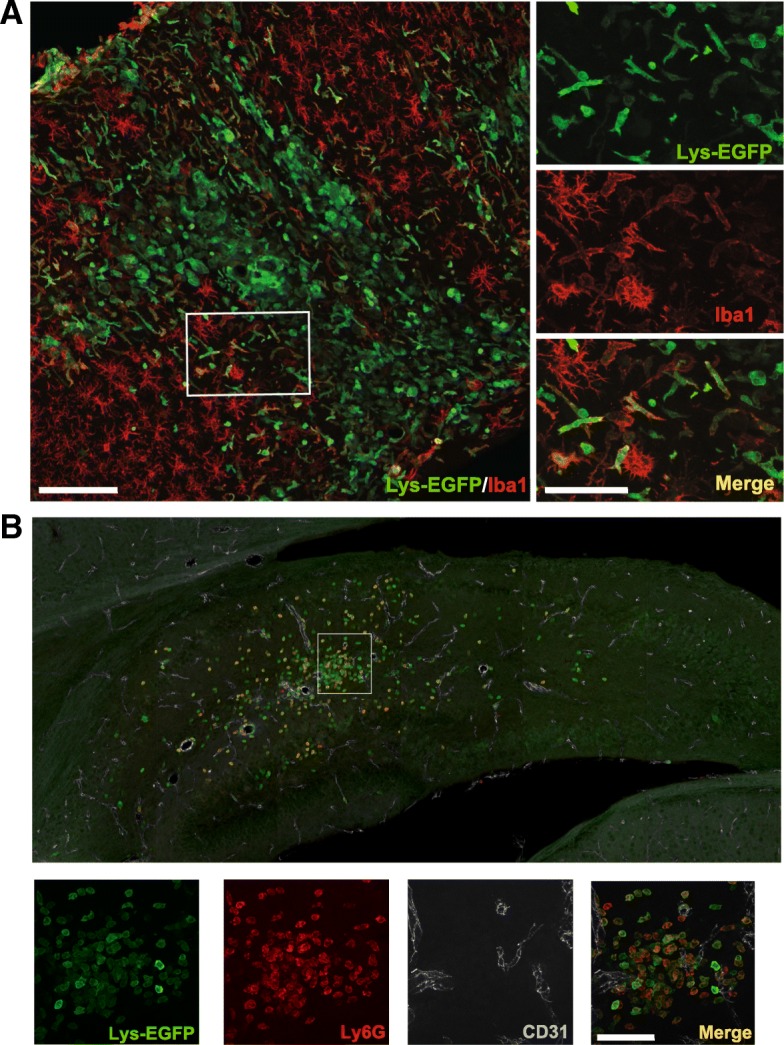
EGFP^+^ myeloid cells in the brain 7 days after hypoxia-ischemia are Iba-1^−^ and Ly6G^+^. **a** EGFP^+^ cells are distinct from Iba-1-positive cells and display mostly round morphology in the ipsilateral cortex in animals with severe injury at 7 days after hypoxia-ischemia (HI). **b** Round-shaped EGFP^+^ cells are largely Ly6G^+^ and are not associated with CD31^+^ vessels (example from the hippocampus). Scale bars = 100 μm (at low magnification) and 50 μm (at high magnification)

Different localisation patterns of infiltrating cells were determined at 1 day and 7 days after HI by immunohistochemical staining for EGFP. We further co-localised EGFP^+^ staining with the endothelial cell marker CD31 at 1 day and 7 days after HI to assess parenchymal vs intraluminal localisation. At 1 day, EGFP^+^ myeloid cells show a dispersed pattern of infiltration including invasion of the hippocampus, thalamus (Fig. 2a) and striatum (Additional file 1: Figure S1A). In contrast, at 7 days, invading cells showed a spatially distinct dense pattern of infiltration limited to the remaining parts of the hippocampus and hippocampal fimbriae as well as the white matter in the thalamus (Fig. 2b), with very little infiltration into the striatum (Additional file 1: Figure S1B). Cortical infiltration was also observed in cases with severe injury at both time points (Fig. 3a).

### Invading EGFP^+^ leukocytes include cell types of distinct morphologies

As *Lys*-EGFP-*ki* transgenic mice express EGFP in monocytes, MDMs and granulocytes [23], we employed confocal microscopy in conjunction with immunohistochemistry to investigate the morphological features and protein immunoreactivity of CNS-infiltrating cells 7 days after HI. In animals with severe injury, EGFP^+^ infiltrating cells were present in the cortex and displayed low to negative immunoreactivity for microglial/monocyte marker Iba1^+^ and were commonly round shaped (Fig. 3a). Similarly, in the injured hippocampus, EGFP^+^ cells were commonly round and expressed the neutrophil marker Ly6G but were not associated with CD31^+^ vessels (Fig. 3b).

### CNS accumulation of inflammatory monocytes and granulocytes after HI follows a temporally biphasic pattern

**Fig. 4:**
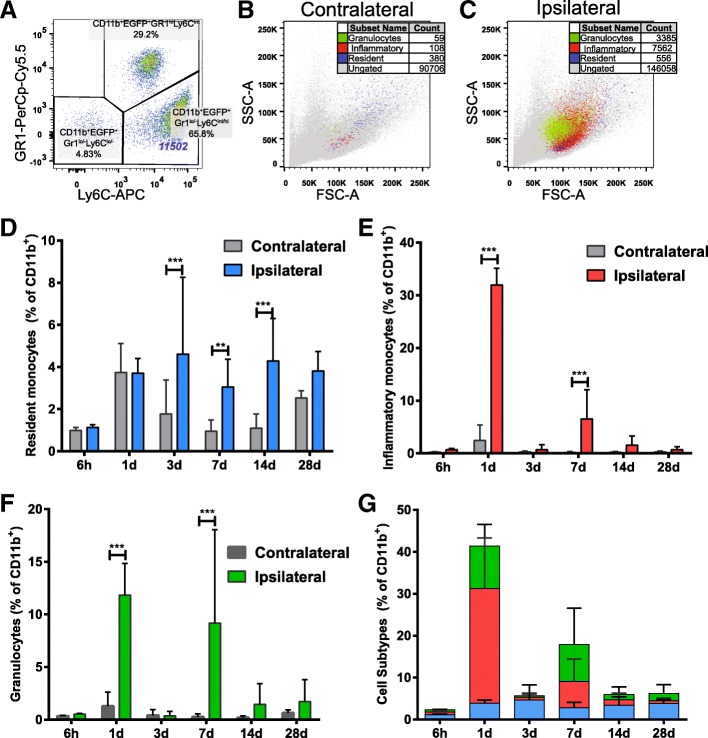
Characterisation of EGFP^+^ cells in the brain after hypoxia-ischemia. EGFP^+^ myeloid cells were identified after hypoxia-ischemia (HI) through the gating strategy presented in Fig. 1a–c. **a** EGFP^+^ were further characterised based on expression of Gr1 and Ly6C and defined as resident monocytes (CD11b^+^EGFP^+^Gr1^lo/−^Ly6C^lo/−^), inflammatory monocytes (CD11b^+^EGFP^+^Gr1^lo/−^Ly6C^int/hi^) and granulocytes (CD11b^+^EGFP^+^Gr1^hi^Ly6C^int^). **b**, **c** Backgating displays the presence of these cell populations in contralateral (**b**) and ipsilateral (**c**) hemispheres at 1 day after HI. **d**–**f** Compiled data demonstrate these cell subtypes in ipsilateral and contralateral hemispheres at 6 h (*n* = 8), 1 day (*n* = 7), 3 days (*n* = 23), 7 days (*n* = 19), 14 days (*n* = 14) and 28 days (*n* = 9) after HI. **g** Stacked bar graph showing the relative contribution of each cell population to the total EGFP^+^ population at each time point. Values are presented as the mean ± SD. One-way ANOVA followed by Holm-Sidak’s post hoc test comparing differences between hemispheres at each time point, **p ≤ 0.01, ***p ≤ 0.001

In order to identify subpopulations of infiltrating CD11b^+^EGFP^+^ leukocytes, cells were gated based on their expression of Gr1 and Ly6C, allowing determination of inflammatory monocytes (CD11b^+^EGFP^+^Gr1^lo/−^Ly6C^int/hi^), resident monocytes (CD11b^+^EGFP^+^Gr1^lo/−^Ly6C^lo/−^) and granulocytes (CD11b^+^EGFP^+^Gr1^hi^Ly6C^int^) [26] (Fig. 4a). Backgating confirmed the relative lack of infiltrating cells in the contralateral hemisphere (Fig. 4b) and the distinct physical properties of each myeloid subpopulation in the ipsilateral hemisphere (Fig. 4c), with granulocytes and resident monocytes forming distinct populations based on size and granularity while inflammatory monocytes formed a less homogenous population.

 CD11b^+^EGFP^+^Gr1^lo/−^Ly6C^lo/−^ resident monocytes could be detected at significantly greater levels in the ipsilateral hemisphere compared to contralateral levels at 3 days (*p* < 0.001), 7 days (*p* < 0.01) and 14 days (*p* < 0.001) after HI (Fig. 4d). Inflammatory monocytes were significantly increased in the ipsilateral compared to contralateral hemisphere at 1 day after HI and represented 31.9 ± 1.3% of the ipsilateral hemisphere’s total CD11b^+^ cell population (Fig. 4e; *p* < 0.001); by 3 days, however, they were no longer detectable above control levels (Fig. 4e, *p* > 0.05). A second phase of infiltration occurred after 3 days with significantly more inflammatory monocytes detected at 7 days (*p* < 0.001) in the ipsilateral compared to the contralateral hemisphere. The infiltration of CD11b^+^EGFP^+^Gr1^hi^Ly6C^int^ granulocytes (Fig. 4f) largely mirrored that of inflammatory monocytes with the increased number of granulocytes observed in the ipsilateral hemisphere at 1 day (*p* < 0.001) and 7 days (*p* < 0.001). Figure 4g displays an overview of the relative contribution of resident monocytes, inflammatory monocytes and granulocytes to the total population of peripherally derived myeloid cells in the ipsilateral hemisphere at each time point (expressed as percentage of total CD11b^+^ cells). Overall, the temporal pattern of peripheral leukocyte influx was distinctly biphasic, characterised by peak accumulation of inflammatory cells (inflammatory monocytes and granulocytes) at 1 day, relative quiescence at 3 days and renewed infiltration at 7 days.

### Inflammatory cell accumulation in the brain is associated with elevated chemotactic and inflammatory cytokine levels

**Fig. 5:**
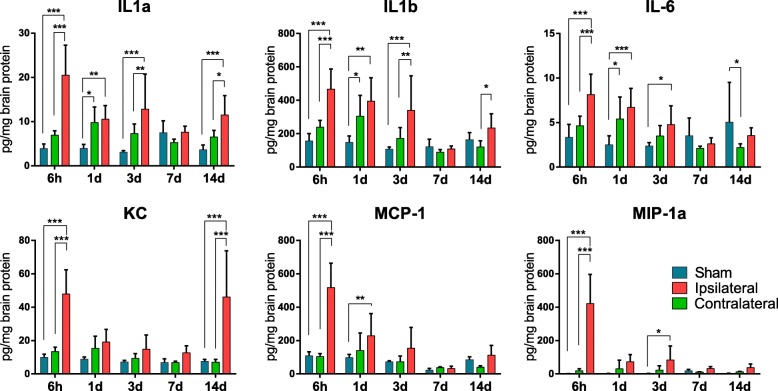
Temporal characterisation of CNS chemo- and cytokine regulation following hypoxia-ischemia. Inflammatory responses in brain tissue were investigated using multiplex analysis at 6 h, 1 day, 3 days, 7 days and 14 days after neonatal hypoxia-ischemia. Values are pg/mg (picogram cytokine per milligram brain protein) and presented as the mean ± SD. One-way ANOVA followed by Holm-Sidak’s post hoc test for comparing the differences between sham-operated mice (blue bar) and contralateral (green bar) and ipsilateral (red bar) hemispheres at each time point. *n* = 5 for sham group and *n* = 8 for HI groups for each time point. *p ≤ 0.05, **p ≤ 0.01, ***p ≤ 0.001

To assess whether the temporal pattern of leukocyte infiltration to the brain corresponded to a distinct cytokine profile, we performed a 23-plex multiplex cytokine assay covering the time points assessed in the flow cytometry experiment. At 6 h, before the peak of leukocyte accumulation, a marked increase was observed in several inflammatory cytokines (e.g. IL1a, IL1b and IL6) and chemokines (e.g. KC, MCP1, MIP1a, MIP1b), as well as granulocyte and macrophage growth factors (e.g. G-CSF and GM-CSF), in the ipsilateral hemisphere compared to the contralateral hemisphere (Fig. 5, Additional file 2: Figure S2, Additional file 3 and Additional file 4: Table S1). Several cytokines (e.g. IL1a, IL1b and IL6; Fig. 5) were also increased in the contralateral hemisphere compared to that in the brains of sham-operated mice. Chemokine levels were similar in the contralateral hemisphere compared with that in the brains from sham-operated mice. Cytokine levels in the ipsilateral hemisphere remained elevated over time, except for the 7-day time point. Most chemokines were reduced to sham levels within 1–3 days and remained at levels similar to those observed in sham-operated animals up to 2 weeks except for KC, which was markedly increased at 14 days after HI (Fig. 5).

Significant differences in plasma cytokine levels between HI mice and sham-operated mice were only detected for G-CSF at 1 day and four cytokines (IL1b, IL3, IL4 and eotaxin) which were elevated at the 14-day time point in HI mice (Additional file 3: Figure S3 and Additional file 5: Table S2).

### Myeloid cell depletion protects the brain against hypoxic-ischemic injury in male mice

**Fig. 6:**
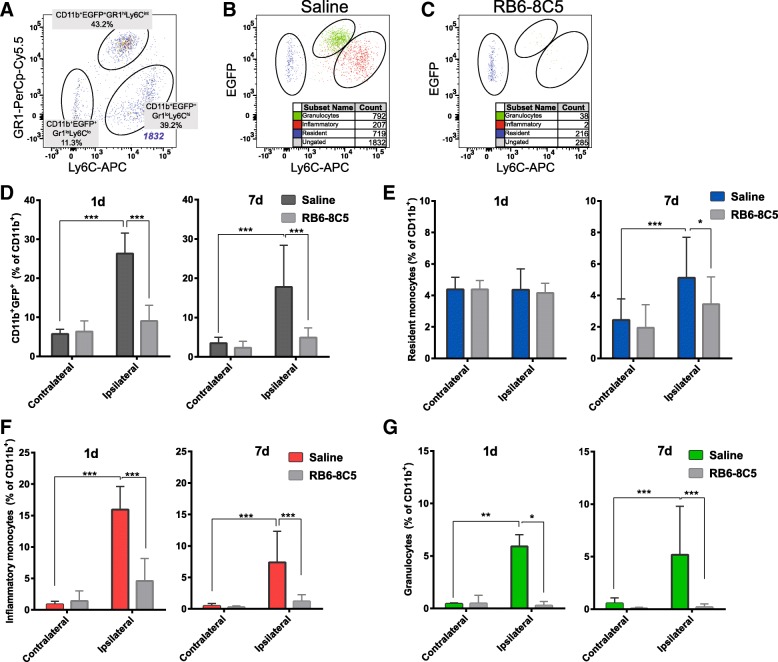
Depletion of myeloid cells in the injured CNS. **a–c** Using the same gating strategy as described in Fig. 1a–c and Fig. 4a, EGFP^+^ cells in the brain after saline injection (**b**) or RB6-8C5 antibody administration (**c**) were identified as resident monocytes (CD11b^+^EGFP^+^Ly6C^lo/−^), inflammatory monocytes (CD11b^+^EGFP^+^Ly6C^hi^) and granulocytes (CD11b^+^EGFP^+^Ly6C^int^). **d**–**g** Compiled data displays the effect of RB6-8C5 antibody administration on the overall CD11b^+^EGFP^+^ cell population (**d**) resident monocytes (**e**), inflammatory monocytes (**f**) and granulocytes (**g**), at 1 day (Sal: *n* = 6; RB6-8C5: *n* = 3) and 7 days (Sal: *n* = 12; RB6-8C5: *n* = 13) after hypoxia-ischemia in the ipsilateral and contralateral hemispheres. Values are presented as mean ± SD. One-way ANOVA followed by Holm-Sidak’s post hoc test was used for comparing the differences between the contralateral and ipsilateral hemispheres at different time points. *p ≤ 0.05, **p ≤ 0.01, ***p ≤ 0.001

To investigate the role of the infiltrating myeloid cells, an antibody-based cell-depleting strategy was employed. Repeated systemic administration of RB6-8C5 blocked accumulation of myeloid cells in the brain after HI (Fig. 6b, c). At 1 day and 7 days after HI, the percentage of CD11b^+^EGFP^+^ infiltrating myeloid cells were significantly lower in the injured hemisphere of antibody-treated mice compared to that in the control mice (Fig. 6d). Likewise, antibody administration significantly reduced the percentage of inflammatory monocytes (CD11b^+^EGFP^+^Ly6C^hi^) and granulocytes (CD11b^+^EGFP^+^Gr1^+^Ly6C^int^) in the injured hemisphere at both time points (Fig. 6f, g). Interestingly, the percentage of resident monocytes was also lower in antibody-treated mice at the 7-day time point (Fig. 6e).

**Fig. 7:**
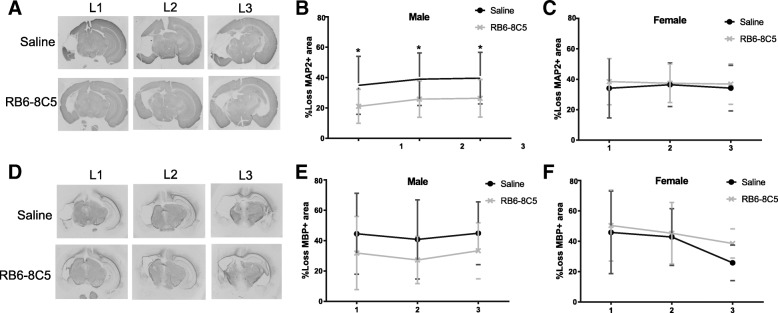
Neuroprotection in males only following myeloid cell depletion. Brain tissue was immuno-labelled for neuronal and white matter tissue using antibodies against MAP2 and MBP, respectively, and tissue loss was measured 14 days after hypoxia-ischemia in mice treated with saline (male *n* = 17; female *n* = 19) or RB6-8C5 antibody (male *n* = 14; female *n* = 20). **a** Representative images of MAP2-stained sections of the brain at three levels (L1–L3) obtained from male mice treated with saline or RB6-8C5 after HI. **b**, **c** RB6-8C5 antibody treatment reduced the MAP2+ neuronal tissue loss in male (**b**) and but not in female (**c**) mice. **d**, **e** Representative images of MBP-stained sections of the brain at three levels (L1–L3) obtained from male mice treated with saline or RB6-8C5 after HI. **e**, **f** RB6-8C5 antibody treatment did not significantly affect the MBP+ myelin loss in male (**e**) and female (**f**) mice. Values are presented as the mean ± SD. Brain injury comparisons were performed using multiple unpaired Student’s *t* tests at each brain level; *p* values were corrected for multiple comparison using the Holm-Sidak method. *p ≤ 0.05

Next, we asked whether inhibition of myeloid cell accumulation in the brain affects the HI injury. Treatment with RB6-8C5 significantly reduced loss of MAP2-positive neuronal tissue in male mice (*p* < 0.05, Fig. 7a, b), but not in female mice (Fig. 7c). There was no significant change in loss of MBP-positive area (i.e. myelinated area) in either male (Fig. 7d, e) or female mice (Fig. 7f).

## Discussion

In this study, we utilised the *Lys-*EGFP*-ki* mouse model, in which myeloid cells generated through definitive but not primitive haematopoiesis express EGFP [23, 28], to characterise the presence of peripherally derived myeloid cells in the injured CNS after neonatal HI. We show that EGFP-positive leukocytes home to the injured hemisphere and are morphologically distinct from CNS microglia and demonstrate a temporally biphasic pattern of inflammatory cell infiltration occurring over a background of stable resident monocyte infiltration. Further, we show that depletion of these cells reduces grey matter injury in male mice.

Myeloid cell accumulation has been demonstrated in numerous neonatal models of sterile brain injury [29] [10, 11, 30–32]. While identification of granulocytes in the CNS is relatively straightforward, discrimination of MDMs and MiDMs has presented a greater problem due to their similar characteristics and common expression of cell surface epitopes [15, 16]. *Lys*-EGFP-*ki* transgenic mice express EGFP in monocytes, MDMs and granulocytes [23] allowing unambiguous identification of CNS-infiltrating cells and therefore discrimination of MDMs and MiDMs by flow cytometry and microscopy [28].

Our immunohistochemical investigation indicated extravasated EGFP^+^ cells as early as 24 h after HI, with cells dispersed throughout the ipsilateral hemisphere. Later at the 7-day time point, there was a close correlation between peripheral myeloid cell accumulation and well-characterised areas of cerebral injury that has previously been described in the neonatal HI model [33]. Microscopic examination of EGFP^+^ cells in the cortex at 7 days revealed distinct morphological characteristics of invading cells, which displayed few processes and low Iba1 immunoreactivity. Although qualitative in nature, this data reflects recent observations made following experimental autoimmune encephalomyelitis in CCR2-RFP/CX3CR1-GFP mice [17], where unlike the highly ramified microglia, MDMs were elongated or spindle shaped, smaller and rarely had processes. EGFP^+^ cells often co-localised with Ly6G^+^ cells but not with CD31^+^ blood vessels (Fig. 3b), supporting that these cells were peripherally derived myeloid cells.

We have previously shown marked expansion of the CD11b^+^ cell population in the brain after neonatal HI [34]. Using quantitative flow cytometry-based analysis of CD11b^+^EGFP^+^ cells, our data indicates that a significant proportion of this CD11b^+^ cell population in the ipsilateral hemisphere consists of infiltrating myeloid cells, ranging from 28 to 48% of the total CD11b^+^ cells at 1 day after HI. The difference in percentage of infiltrating cells between experiments is likely due to the variability in the HI model. However, overall, our data indicates a larger response than that observed by Denker et al. in a neonatal stroke model where accumulation of 10% CD45^hi^CD11b^+^ cells was observed at 1 day after MCAO [32]. The discrepancies in the different models may result from the utilisation of different strategies for the identification of infiltrating leukocytes, inherent differences of the rat neonatal stroke and mouse HI models, but also variability in respective models.

Further characterisation of the CD11b^+^EGFP^+^ cell populations based on differential expression of Ly6C and Gr1 facilitated discrimination of resident monocytes (CD11b^+^EGFP^+^Gr1^lo/−^Ly6C^lo/−^), inflammatory monocytes (CD11b^+^EGFP^+^Gr1^lo/−^Ly6C^int/hi^) [25] and granulocytes (CD11b^+^EGFP^+^Gr1^hi^Ly6C^int^) [26]. The two pro-inflammatory cell types identified, inflammatory monocytes and granulocytes, displayed temporally similar expression patterns with peak accumulation seen at 1 and 7 days after HI but with no significant increase at 3 days and a minimal but detectable presence at 14 days, suggesting distinct phases of accumulation and clearance.

Previous studies examining neutrophil accumulation after neonatal HI reported varying results. Several studies demonstrated neuroprotective effects by depleting neutrophils before HI [12, 13, 35], suggesting an important role of neutrophils in injury development. One of these studies, however, was unable to show consistent hemispheric differences in myeloperoxidase (MPO) activity, number of MPO positive cells and number of anti-neutrophil serum-positive cells [12]. Nijboer et al. demonstrated increased MPO activity at 24 h and up to 48 h [13], and identification and quantification of neutrophils in H&E-stained sections revealed increased neutrophil counts at 12 h, but not at 24 h–35 days [11]. Our data complements the above described studies by displaying increased post-ischemia granulocyte accumulation as assessed in an unbiased and quantitative fashion.

The distinct peaks of inflammatory cell accumulation detected at 1 day and 7 days suggest distinct phases of infiltration and clearance, presumably by CNS resident macrophages. These observations are partially supported by previous studies: Winerdal et al. demonstrated peak CD11b^+^CD86^+^ macrophage presence at 1 day and 7 days, with reduced levels at 3 days after neonatal HI in mice [36], and in adult rats, distinct peaks of neutrophil engulfment by macrophages were observed 3 days and 15 days post-ischemia [37]. Speculatively, these studies suggest distinct phases of influx and phagocytic clearance of infiltrating inflammatory cells. Resident monocytes, by contrast, were detected at lower and more stable levels. This pattern of infiltration may be due to a slower accumulation of resident monocytes coupled with a lesser degree of phagocytic clearance: a fate that is in light of the proposed reparatory phenotype of these cells [20, 38].

Previous microarray data from our laboratory indicated peak expression of several chemokines in the brain, including CCL2, CCL7, CCL3 and CXCL1 at 8 h post-HI, with reduced levels at 3 days (the latest time point assessed), presumably produced by endogenous brain cells [8]. Thus, we now hypothesised that increased chemokine expression in the brain could attract peripheral immune cells. However, the multiplex analyses conducted in this study failed to demonstrate a correlation between elevated cytokine and chemokine levels and the temporal pattern of leukocyte accumulation. This was particularly apparent at the early time points where marked increases in chemokine levels were observed at 6 h, while peak leukocyte accumulation occurred at 24 h after HI. These data thus suggest that increased chemokine expression precedes cell accumulation. Hence, by the time significant cell accumulation can be detected, the chemokine gradient might have already started to fade away. In support, there was also no evidence of cytokine and chemokine changes in the brain at 7 days after HI, when the second phase of leukocyte accumulation was identified. However, we cannot exclude the possibility that a second wave of infiltration was due to cytokine/chemokine gradients between days 3 and 7. The lack of correlation between CNS inflammation and infiltrating cells could also be due to that we only investigated the overall response in the whole brain. It is possible that specific cell types/brain structures, for example the response in the vasculature, are more directly correlated to outcome. Further, interestingly, a number of cytokines/chemokines were increased in the brain even 14 days after HI. For example, KC showed a biphasic pattern of increase after HI, with the first and second phase at 6 h and 14 days after HI, respectively. It is unclear how this long-term CNS inflammation may have affected outcome. Previous studies using the neonatal HI model have shown a progressive cerebral atrophy which is only apparent from approximately 2 weeks after the initial injury [39]. Thus, it would be of interest to, in future experiments, investigate both inflammatory responses and immune cell infiltration over a more prolonged period of time and in specific brain/vascular structures.

In addition to chemokine gradients, the physiological status of the brain barriers may affect the magnitude of the leukocyte accumulation. However, we have previously shown an increase in blood-brain barrier permeability at 6 h and 24 h, but not at 7 days following neonatal HI [40]. These results suggest that other mechanisms are likely to be involved in the second wave of leukocyte accumulation. The choroid plexus has been shown to be the route of entry for T_H_17 lymphocytes into the brain following inflammation-sensitised neonatal HI [38]. We recently showed that following systemic inflammation, induced by a toll-like receptor 2 agonist, marked leukocyte infiltration occurred through the choroid plexus [41]. Thus, it is possible that the trafficking across the choroid plexus may have contributed to the second wave of leukocyte accumulation.

Recruitment of peripheral neutrophils, monocytes or lymphocytes aggravates the damage in animal models of multiple sclerosis [42, 43], stroke [37, 44, 45] and epilepsy [46]. We have demonstrated that systemic activation of toll-like receptor 2 induces infiltration of leukocytes, mainly neutrophils and inflammatory monocytes, to the developing brain of mice and exacerbates HI brain injury [41, 47]. In contrast, a previous study in adult animals reported that a subtype population of monocytes homes to the injury site via the choroid plexus following spinal cord injury and contributes to the resolution of inflammation [48]. Therefore, current evidence suggests that invading leukocytes can play both detrimental and beneficial roles depending on their subtype, the nature of the injury and potentially age.

We found that the systemic depletion of neutrophil and monocyte populations inhibits their accumulation in the brain following HI and reduces the neuronal injury in males. The role of inflammatory cells in ischemic injury of the brain is controversial [49]. Recently, accumulation of neutrophils in the human brain after adult stroke has been documented [50], supported by similar observation in a mouse model of stroke [50]. Moreover, depletion of neutrophils has been shown to reduce the infarct size in a rat model of brain ischemic injury [51]. However, in a focal ischemic stroke rat model, depletion of neutrophils failed to reduce the brain injury [52]. Our results, particularly the gender-dependent aspect, corroborate the complex role leukocytes play in ischemic injuries. Accumulating evidence illustrates gender-related differences in immune responses to infection and injury in adults and newborns, which was attributed to effects of various sex hormones as well as of differences on the sex chromosome [53]. In adult mice, induction of certain cytokines in the brain was stronger in males in systemic inflammation models and in an experimental autoimmune encephalomyelitis model [54, 55]. In neonatal mice (P4), there are more microglia colonised in the brain of males than in females [56]. Very recently, it was discovered that microglial response to environmental challenges is sex-specific [57]. There are also differences between male and female in the quantity and function of neutrophils and monocytes [58–61]. Further, inflammatory responses and injury outcome after neonatal HI have been reported to diverge according to gender, with enhanced inflammation, microglia activation and monocyte infiltration in males compared to females [62, 63]. Therefore, it is not surprising that many neuroprotective agents for neonatal brain injury, such as the antibody treatment in the present study, act in a sex-specific manner [64–67].

In conclusion, we have assessed accumulation of peripherally derived myeloid cells in the immature CNS in response to experimental HI. We detected significant infiltration with highest levels of invading cells at 1 day after hypoxia-ischemia. Additionally, we demonstrate two distinct phases of inflammatory cell accumulation occurring over a background of stable resident monocyte accumulation. Moreover, inhibiting myeloid cell accumulation in the brain was shown to be neuroprotective in male mice. Our findings merit renewed interest in the roles of MDMs and polymorphonuclear leukocytes in the long-term aspects of neonatal brain injury.

## Conclusion

By using *Lys*-EGFP-*ki* mice, allowing identification of peripheral myeloid cells in the brain, we demonstrate a temporally biphasic pattern of inflammatory monocyte and granulocyte infiltration after neonatal hypoxia-ischemia. Antibody-mediated depletion of circulating myeloid cells reduced immune cell accumulation in the brain and reduced neuronal loss in male but not female mice. This study offers new insights into sex-dependent central-peripheral immune communication following neonatal brain injury and merits renewed interest in the roles of granulocytes and monocytes in brain lesion development.

## Additional files

Additional file 1: **Figure S1.** EGFP^+^ myeloid cell localisation in the brain after HI. Representative tile-scanned confocal images of brain sections after HI. EGFP^+^ myeloid cells markedly infiltrate the striatum 1 day (A) but not 7 days (B) after HI. *n* = 4. (PDF 7477 kb)

Additional file 2: **Figure S2.** Cytokine measurements in the brain after HI. Multiplex cytokine measurements in the brain at 6 h, 1 day, 3 days, 7 days and 14 days after neonatal hypoxia-ischemia (HI) or sham operation. Values are pg/mg protein and presented as the mean ± SD. The statistical results are presented in Additional file 4: Table S1. (PDF 172 kb)

Additional file 3: **Figure S3**. Cytokine measurements in plasma after HI. Multiplex cytokine measurement in plasma at 6 h, 1 day, 3 days, 7 days and 14 days after neonatal hypoxia-ischemia (HI) or sham operation. Values are pg/ml and presented as the mean ± SD. The statistical results are presented in Additional file 5: Table S2. (PDF 172 kb)

Additional file 4: **Table S1.** Statistical analysis of multiplex cytokine measurement in the brain after HI. One-way ANOVA followed by Holm-Sidak’s post hoc test for comparing the differences between sham-operated mice and HI mice in the contralateral and ipsilateral hemispheres at each time point. *n* = 5 for sham group and *n* = 8 for HI groups. ns: not significant, *p < 0.05, **p < 0.01, ***p < 0.001. (PDF 138 kb)

Additional file 5: **Table S2.** Statistical analysis of multiplex cytokine measurement in plasma after HI. Student’s *t* test followed by Holm-Sidak’s post hoc test to correct for multiple comparisons between sham-operated mice and HI mice at each time point. *n* = 5 for sham group and *n* = 8 for HI groups. ns: not significant, *p < 0.05. (PDF 164 kb)

## Abbreviations

BSA: Bovine albumin serum
CNS: Central nervous system
EGFP: Enhanced green fluorescent protein
HBSS: Hanks’ Balanced Salt Solution
HI: Hypoxia-ischemia
MAP 2: Microtubule-associated protein-2
MBP: Myelin basic protein
MDM: Monocyte-derived macrophages
MiDM: Microglial-derived macrophages
MPO: Myeloperoxidase
P: Postnatal day
PFA: Paraformaldehyde
TBS: Tris-buffered saline

## References

[1] Hagberg H, Mallard C, Ferriero DM, Vannucci SJ, Levison SW, Vexler ZS, Gressens P (2015). The role of inflammation in perinatal brain injury. Nat Rev Neurol.

[2] Degos V, Favrais G, Kaindl AM, Peineau S, Guerrot AM, Verney C, Gressens P (2010). Inflammation processes in perinatal brain damage. J Neural Transm.

[3] Bilbo SD, Schwarz JM (2012). The immune system and developmental programming of brain and behavior. Front Neuroendocrinol.

[4] Hagberg H, Gressens P, Mallard C (2012). Inflammation during fetal and neonatal life: implications for neurologic and neuropsychiatric disease in children and adults. Ann Neurol.

[5] Ginhoux F, Greter M, Leboeuf M, Nandi S, See P, Gokhan S, Mehler MF, Conway SJ, Ng LG, Stanley ER (2010). Fate mapping analysis reveals that adult microglia derive from primitive macrophages. Science.

[6] Galea I, Bechmann I, Perry VH (2007). What is immune privilege (not)?. Trends Immunol.

[7] Ransohoff RM, Kivisakk P, Kidd G (2003). Three or more routes for leukocyte migration into the central nervous system. Nat Rev Immunol.

[8] Hedtjarn M, Mallard C, Hagberg H (2004). Inflammatory gene profiling in the developing mouse brain after hypoxia-ischemia. J Cereb Blood Flow Metab.

[9] Shi C, Pamer EG (2011). Monocyte recruitment during infection and inflammation. Nat Rev Immunol.

[10] McRae A, Gilland E, Bona E, Hagberg H (1995). Microglia activation after neonatal hypoxic-ischemia. Brain Res Dev Brain Res.

[11] Bona E, Andersson AL, Blomgren K, Gilland E, Puka-Sundvall M, Gustafson K, Hagberg H (1999). Chemokine and inflammatory cell response to hypoxia-ischemia in immature rats. Pediatr Res.

[12] Hudome S, Palmer C, Roberts RL, Mauger D, Housman C, Towfighi J (1997). The role of neutrophils in the production of hypoxic-ischemic brain injury in the neonatal rat. Pediatr Res.

[13] Nijboer CH, Kavelaars A, Vroon A, Groenendaal F, van Bel F, Heijnen CJ (2008). Low endogenous G-protein-coupled receptor kinase 2 sensitizes the immature brain to hypoxia-ischemia-induced gray and white matter damage. J Neurosci.

[14] Jin Y, Silverman AJ, Vannucci SJ (2009). Mast cells are early responders after hypoxia-ischemia in immature rat brain. Stroke.

[15] Perry VH, Hume DA, Gordon S (1985). Immunohistochemical localization of macrophages and microglia in the adult and developing mouse brain. Neuroscience.

[16] Sedgwick JD, Schwender S, Imrich H, Dorries R, Butcher GW, ter Meulen V (1991). Isolation and direct characterization of resident microglial cells from the normal and inflamed central nervous system. Proc Natl Acad Sci U S A.

[17] Yamasaki R, Lu H, Butovsky O, Ohno N, Rietsch AM, Cialic R, Wu PM, Doykan CE, Lin J, Cotleur AC (2014). Differential roles of microglia and monocytes in the inflamed central nervous system. J Exp Med.

[18] Geissmann F, Jung S, Littman DR (2003). Blood monocytes consist of two principal subsets with distinct migratory properties. Immunity.

[19] Mildner A, Mack M, Schmidt H, Bruck W, Djukic M, Zabel MD, Hille A, Priller J, Prinz M (2009). CCR2+Ly-6Chi monocytes are crucial for the effector phase of autoimmunity in the central nervous system. Brain.

[20] Auffray C, Fogg D, Garfa M, Elain G, Join-Lambert O, Kayal S, Sarnacki S, Cumano A, Lauvau G, Geissmann F (2007). Monitoring of blood vessels and tissues by a population of monocytes with patrolling behavior. Science.

[21] Gliem M, Mausberg AK, Lee JI, Simiantonakis I, van Rooijen N, Hartung HP, Jander S (2012). Macrophages prevent hemorrhagic infarct transformation in murine stroke models. Ann Neurol.

[22] Yang Jiyeon, Zhang Lixiao, Yu Caijia, Yang Xiao-Feng, Wang Hong (2014). Monocyte and macrophage differentiation: circulation inflammatory monocyte as biomarker for inflammatory diseases. Biomarker Research.

[23] Faust N, Varas F, Kelly LM, Heck S, Graf T (2000). Insertion of enhanced green fluorescent protein into the lysozyme gene creates mice with green fluorescent granulocytes and macrophages. Blood.

[24] Thawer SG, Mawhinney L, Chadwick K, de Chickera SN, Weaver LC, Brown A, Dekaban GA (2013). Temporal changes in monocyte and macrophage subsets and microglial macrophages following spinal cord injury in the Lys-Egfp-ki mouse model. J Neuroimmunol.

[25] Geissmann F, Manz MG, Jung S, Sieweke MH, Merad M, Ley K (2010). Development of monocytes, macrophages, and dendritic cells. Science.

[26] Hestdal K, Ruscetti FW, Ihle JN, Jacobsen SE, Dubois CM, Kopp WC, Longo DL, Keller JR (1991). Characterization and regulation of RB6-8C5 antigen expression on murine bone marrow cells. J Immunol.

[27] Daley JM, Thomay AA, Connolly MD, Reichner JS, Albina JE (2008). Use of Ly6G-specific monoclonal antibody to deplete neutrophils in mice. J Leukoc Biol.

[28] Mawhinney LA, Thawer SG, Lu WY, Rooijen N, Weaver LC, Brown A, Dekaban GA (2012). Differential detection and distribution of microglial and hematogenous macrophage populations in the injured spinal cord of lys-EGFP-ki transgenic mice. J Neuropathol Exp Neurol.

[29] Dommergues MA, Plaisant F, Verney C, Gressens P (2003). Early microglial activation following neonatal excitotoxic brain damage in mice: a potential target for neuroprotection. Neuroscience.

[30] Tahraoui SL, Marret S, Bodenant C, Leroux P, Dommergues MA, Evrard P, Gressens P (2001). Central role of microglia in neonatal excitotoxic lesions of the murine periventricular white matter. Brain Pathol.

[31] Derugin N, Wendland M, Muramatsu K, Roberts TP, Gregory G, Ferriero DM, Vexler ZS (2000). Evolution of brain injury after transient middle cerebral artery occlusion in neonatal rats. Stroke.

[32] Denker SP, Ji S, Dingman A, Lee SY, Derugin N, Wendland MF, Vexler ZS (2007). Macrophages are comprised of resident brain microglia not infiltrating peripheral monocytes acutely after neonatal stroke. J Neurochem.

[33] Towfighi J, Zec N, Yager J, Housman C, Vannucci RC (1995). Temporal evolution of neuropathologic changes in an immature rat model of cerebral hypoxia: a light microscopic study. Acta Neuropathol.

[34] Hellstrom Erkenstam N, Smith PL, Fleiss B, Nair S, Svedin P, Wang W, Bostrom M, Gressens P, Hagberg H, Brown KL (2016). Temporal characterization of microglia/macrophage phenotypes in a mouse model of neonatal hypoxic-ischemic brain injury. Front Cell Neurosci.

[35] Palmer C, Roberts RL, Young PI (2004). Timing of neutrophil depletion influences long-term neuroprotection in neonatal rat hypoxic-ischemic brain injury. Pediatr Res.

[36] Winerdal M, Winerdal ME, Kinn J, Urmaliya V, Winqvist O, Aden U (2012). Long lasting local and systemic inflammation after cerebral hypoxic ischemia in newborn mice. PLoS One.

[37] Weston RM, Jones NM, Jarrott B, Callaway JK (2007). Inflammatory cell infiltration after endothelin-1-induced cerebral ischemia: histochemical and myeloperoxidase correlation with temporal changes in brain injury. J Cereb Blood Flow Metab.

[38] Yang D, Sun YY, Bhaumik SK, Li Y, Baumann JM, Lin X, Zhang Y, Lin SH, Dunn RS, Liu CY (2014). Blocking lymphocyte trafficking with FTY720 prevents inflammation-sensitized hypoxic-ischemic brain injury in newborns. J Neurosci.

[39] Geddes R, Vannucci RC, Vannucci SJ (2001). Delayed cerebral atrophy following moderate hypoxia-ischemia in the immature rat. Dev Neurosci.

[40] Ek CJ, D'Angelo B, Baburamani AA, Lehner C, Leverin AL, Smith PL, Nilsson H, Svedin P, Hagberg H, Mallard C (2015). Brain barrier properties and cerebral blood flow in neonatal mice exposed to cerebral hypoxia-ischemia. J Cereb Blood Flow Metab.

[41] Mottahedin A, Smith PL, Hagberg H, Ek CJ, Mallard C (2017). TLR2-mediated leukocyte trafficking to the developing brain. J Leukoc Biol.

[42] Liu L, Belkadi A, Darnall L, Hu T, Drescher C, Cotleur AC, Padovani-Claudio D, He T, Choi K, Lane TE (2010). CXCR2-positive neutrophils are essential for cuprizone-induced demyelination: relevance to multiple sclerosis. Nat Neurosci.

[43] Ajami B, Bennett JL, Krieger C, McNagny KM, Rossi FM (2011). Infiltrating monocytes trigger EAE progression, but do not contribute to the resident microglia pool. Nat Neurosci.

[44] Liesz A, Zhou W, Mracsko E, Karcher S, Bauer H, Schwarting S, Sun L, Bruder D, Stegemann S, Cerwenka A (2011). Inhibition of lymphocyte trafficking shields the brain against deleterious neuroinflammation after stroke. Brain.

[45] Lee S, Chu HX, Kim HA, Real NC, Sharif S, Fleming SB, Mercer AA, Wise LM, Drummond GR, Sobey CG (2015). Effect of a broad-specificity chemokine-binding protein on brain leukocyte infiltration and infarct development. Stroke.

[46] Zattoni M, Mura ML, Deprez F, Schwendener RA, Engelhardt B, Frei K, Fritschy JM (2011). Brain infiltration of leukocytes contributes to the pathophysiology of temporal lobe epilepsy. J Neurosci.

[47] Mottahedin A, Svedin P, Nair S, Mohn CJ, Wang X, Hagberg H, Ek J, Mallard C. Systemic activation of Toll-like receptor 2 suppresses mitochondrial respiration and exacerbates hypoxic-ischemic injury in the developing brain. J Cereb Blood Flow Metab. 2017:271678X17691292. 10.1177/0271678X17691292 PMC5453473 28139935

[48] Shechter R, London A, Schwartz M (2013). Orchestrated leukocyte recruitment to immune-privileged sites: absolute barriers versus educational gates. Nat Rev Immunol.

[49] Jin R, Yang G, Li G (2010). Inflammatory mechanisms in ischemic stroke: role of inflammatory cells. J Leukoc Biol.

[50] Perez-de-Puig I, Miro-Mur F, Ferrer-Ferrer M, Gelpi E, Pedragosa J, Justicia C, Urra X, Chamorro A, Planas AM (2015). Neutrophil recruitment to the brain in mouse and human ischemic stroke. Acta Neuropathol.

[51] Matsuo Y, Onodera H, Shiga Y, Nakamura M, Ninomiya M, Kihara T, Kogure K (1994). Correlation between myeloperoxidase-quantified neutrophil accumulation and ischemic brain injury in the rat. Effects of neutrophil depletion. Stroke.

[52] Harris AK, Ergul A, Kozak A, Machado LS, Johnson MH, Fagan SC (2005). Effect of neutrophil depletion on gelatinase expression, edema formation and hemorrhagic transformation after focal ischemic stroke. BMC Neurosci.

[53] Klein SL, Flanagan KL (2016). Sex differences in immune responses. Nat Rev Immunol.

[54] Posillico C, Speirs I, Tronson N (2017). Sex differences in the hippocampal cytokine response following systemic lipopolysaccharide. Brain Behavior and Immunity.

[55] Russi AE, Ebel ME, Yang Y, Brown MA (2018). Male-specific IL-33 expression regulates sex-dimorphic EAE susceptibility. Proc Natl Acad Sci U S A.

[56] Schwarz JM, Sholar PW, Bilbo SD (2012). Sex differences in microglial colonization of the developing rat brain. J Neurochem.

[57] Thion MS, Low D, Silvin A, Chen J, Grisel P, Schulte-Schrepping J, Blecher R, Ulas T, Squarzoni P, Hoeffel G (2018). Microbiome influences prenatal and adult microglia in a sex-specific manner. Cell.

[58] Pace S, Rossi A, Krauth V, Dehm F, Troisi F, Bilancia R, Weinigel C, Rummler S, Werz O, Sautebin L (2017). Sex differences in prostaglandin biosynthesis in neutrophils during acute inflammation. Sci Rep.

[59] Bain BJ, England JM (1975). Normal haematological values: sex difference in neutrophil count. Br Med J.

[60] Spitzer JA, Zhang P (1996). Gender differences in neutrophil function and cytokine-induced neutrophil chemoattractant generation in endotoxic rats. Inflammation.

[61] Jiang Wei, Gilkeson Gary (2014). Sex differences in monocytes and TLR4 associated immune responses; implications for systemic lupus erythematosus (SLE). Journal of Immunotherapy Applications.

[62] Hill CA, Fitch RH (2012). Sex differences in mechanisms and outcome of neonatal hypoxia-ischemia in rodent models: implications for sex-specific neuroprotection in clinical neonatal practice. Neurol Res Int.

[63] Mirza MA, Ritzel R, Xu Y, McCullough LD, Liu F (2015). Sexually dimorphic outcomes and inflammatory responses in hypoxic-ischemic encephalopathy. J Neuroinflammation.

[64] Nijboer CH, Groenendaal F, Kavelaars A, Hagberg HH, van Bel F, Heijnen CJ (2007). Gender-specific neuroprotection by 2-iminobiotin after hypoxia-ischemia in the neonatal rat via a nitric oxide independent pathway. J Cereb Blood Flow Metab.

[65] Nie X, Lowe DW, Rollins LG, Bentzley J, Fraser JL, Martin R, Singh I, Jenkins D (2016). Sex-specific effects of N-acetylcysteine in neonatal rats treated with hypothermia after severe hypoxia-ischemia. Neurosci Res.

[66] Rodriguez-Fanjul J, Duran Fernandez-Feijoo C, Lopez-Abad M, Lopez Ramos MG, Balada Caballe R, Alcantara-Horillo S, Camprubi Camprubi M (2017). Neuroprotection with hypothermia and allopurinol in an animal model of hypoxic-ischemic injury: is it a gender question?. PLoS One.

[67] Fleiss B, Nilsson MK, Blomgren K, Mallard C (2012). Neuroprotection by the histone deacetylase inhibitor trichostatin A in a model of lipopolysaccharide-sensitised neonatal hypoxic-ischaemic brain injury. J Neuroinflammation.

