# Supplementary material for: Peripheral myeloid cells contribute to brain injury in male neonatal mice
Source: J Neuroinflammation. 2018 Oct 30;15:301. doi: 10.1186/s12974-018-1344-9 (PMC6208095; doi:10.1186/s12974-018-1344-9)
Supplement: Supplementary file 1 — Figure S1. EGFP+ myeloid cell localisation in the brain after HI. Representative tile-scanned confocal images of brain sections after HI. EGFP+ myeloid cells markedly infiltrate the striatum 1 day (A) but not 7 days (B) after HI. n = 4. (PDF 7477 kb) [file 12974_2018_1344_MOESM1_ESM.pdf]

A

B

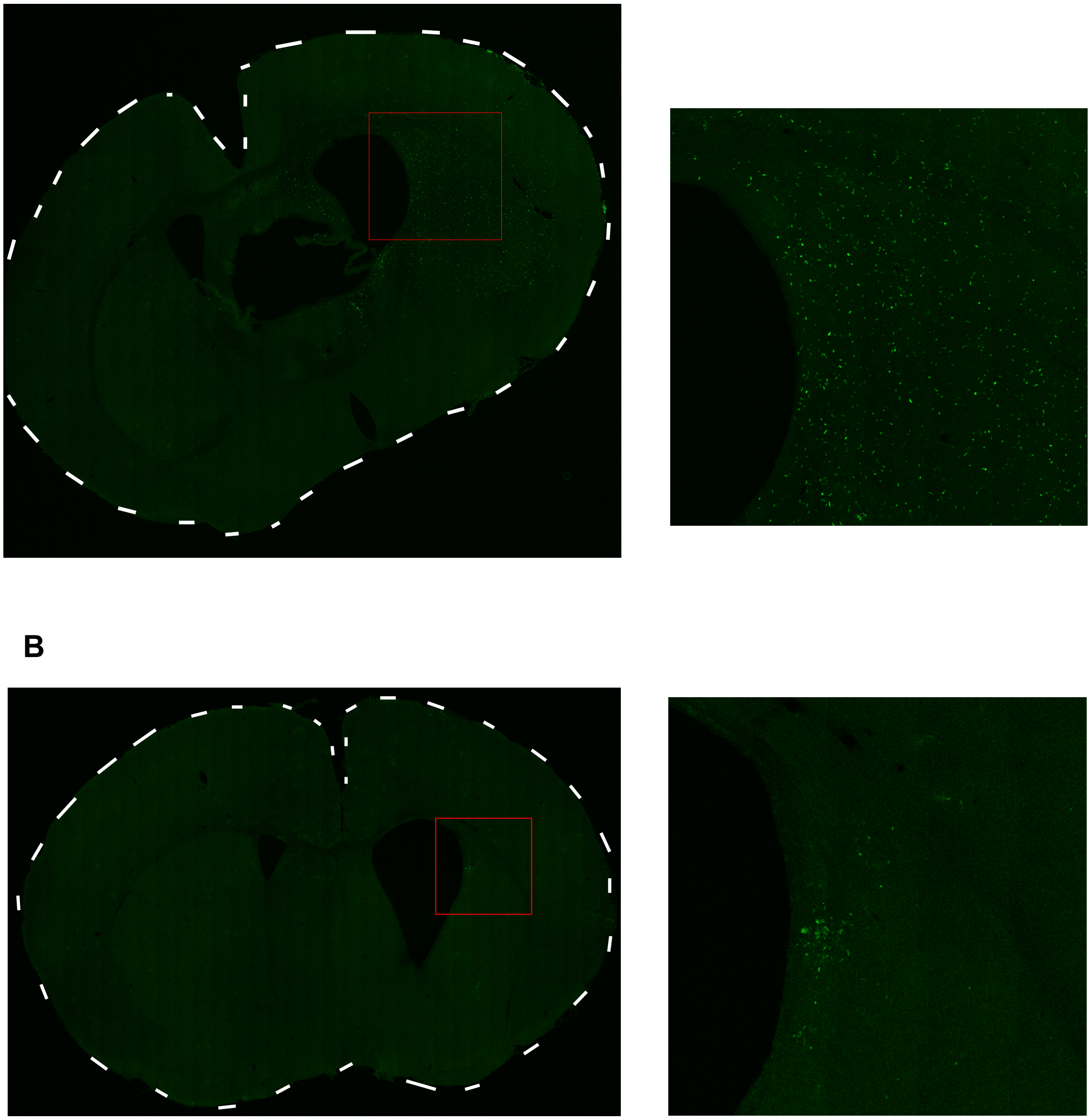

Supplementary figure 1. EGFP+ myeloid cells localization in the brain after HI
Representative tile-scanned confocal images of brain sections after HI. EGFP+ myeloid cells markedly infiltrate the striatum 1 day **(A)** but not 7 day **(B)** after HI. n=4.
