# Supplementary material for: Peripheral myeloid cells contribute to brain injury in male neonatal mice
Source: J Neuroinflammation. 2018 Oct 30;15:301. doi: 10.1186/s12974-018-1344-9 (PMC6208095; doi:10.1186/s12974-018-1344-9)
Supplement: Supplementary file 2 — Figure S2. Cytokine measurements in the brain after HI. Multiplex cytokine measurements in the brain at 6 h, 1 day, 3 days, 7 days and 14 days after neonatal hypoxia-ischemia (HI) or sham operation. Values are pg/mg protein and presented as the mean ± SD. The statistical results are presented in Additional file 4: Table S1. (PDF 172 kb) [file 12974_2018_1344_MOESM2_ESM.pdf]

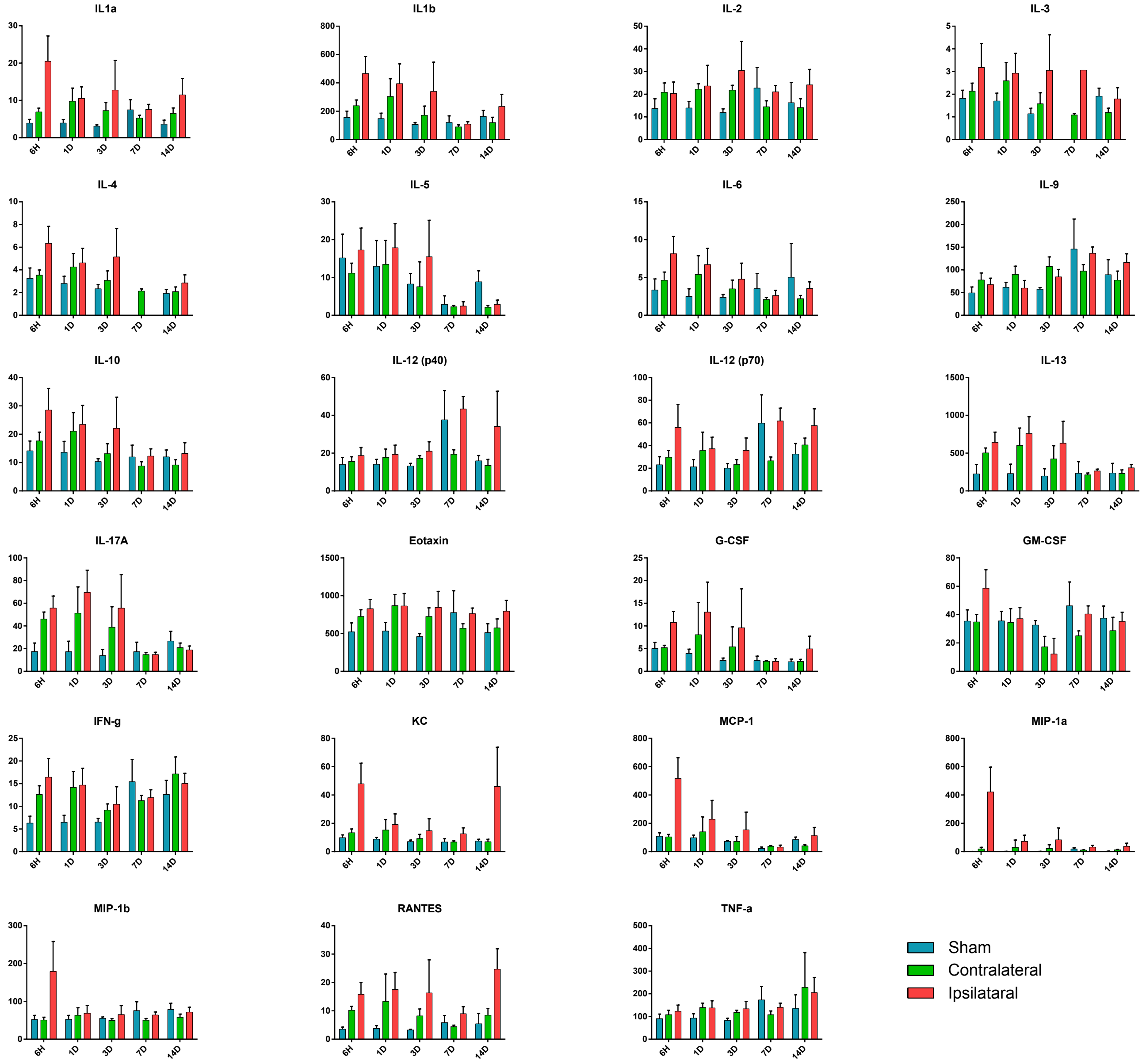

Supplementary figure 2. Multiplex cytokine measurerment in brain at 6 h, 1 d, 3 d, 7 d and 14 d after neonatal hypoxia-ischemia or sham operation. Values are pg/mg protein and presented as the mean ± SD. The statistical results are presented in supplementary table 1.
