# Supplementary material for: Peripheral myeloid cells contribute to brain injury in male neonatal mice
Source: J Neuroinflammation. 2018 Oct 30;15:301. doi: 10.1186/s12974-018-1344-9 (PMC6208095; doi:10.1186/s12974-018-1344-9)
Supplement: Supplementary file 3 — Figure S3. Cytokine measurements in plasma after HI. Multiplex cytokine measurement in plasma at 6 h, 1 day, 3 days, 7 days and 14 days after neonatal hypoxia-ischemia (HI) or sham operation. Values are pg/ml and presented as the mean ± SD. The statistical results are presented in Additional file 5: Table S2. (PDF 172 kb) [file 12974_2018_1344_MOESM3_ESM.pdf]

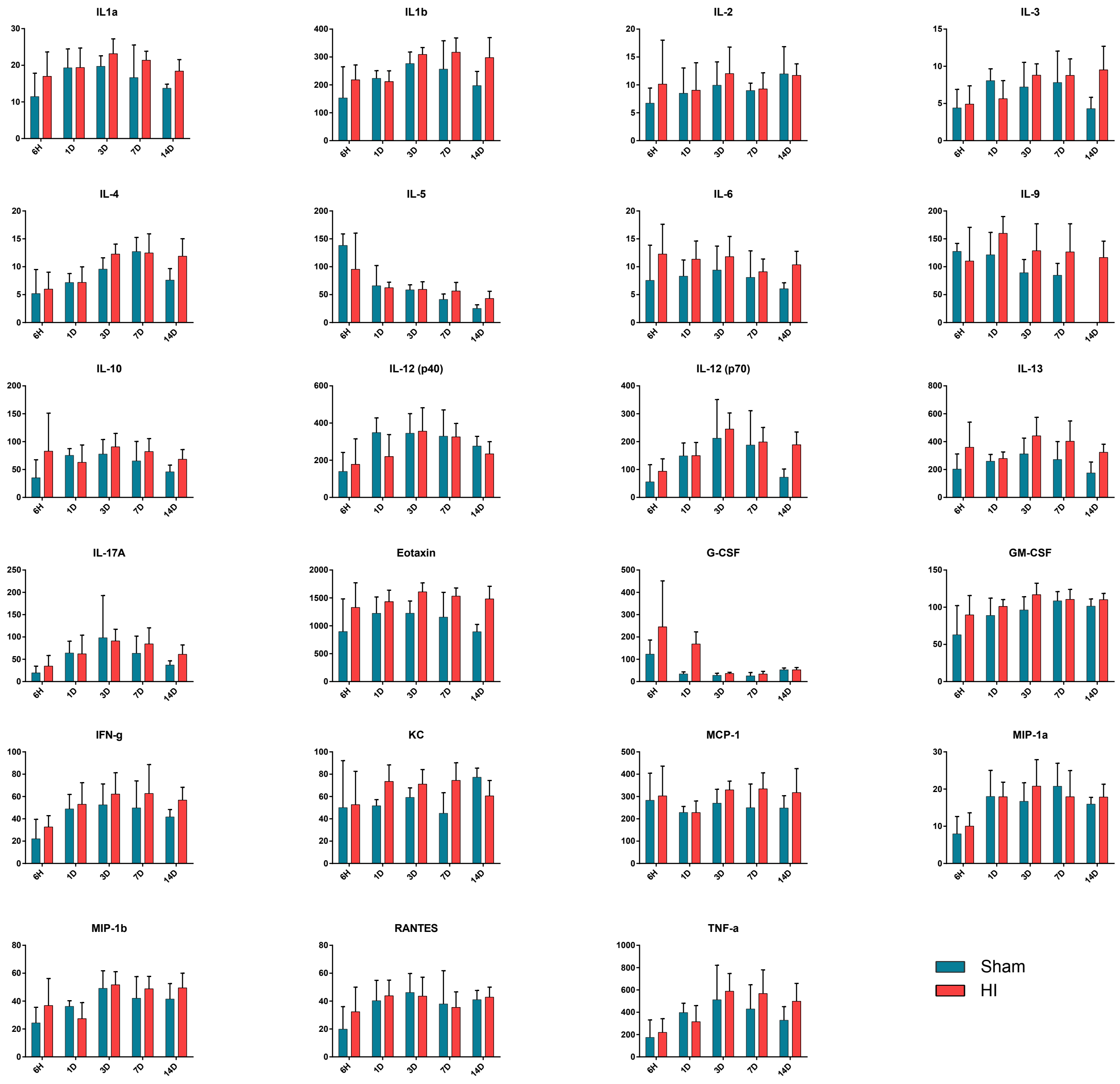

Supplementary figure 3. Multiplex cytokine measurement in plamsa at 6 h, 1 d, 3 d, 7 d and 14 d after neonatal hypoxia-ischemia or sham operation. Values are pg/ml and presented as the mean ± SD. The statistical results are presented in supplementary table 2.
