# Supplementary material for: Peripheral myeloid cells contribute to brain injury in male neonatal mice
Source: J Neuroinflammation. 2018 Oct 30;15:301. doi: 10.1186/s12974-018-1344-9 (PMC6208095; doi:10.1186/s12974-018-1344-9)
Supplement: Supplementary file 4 — Table S1. Statistical analysis of multiplex cytokine measurement in the brain after HI. One-way ANOVA followed by Holm-Sidak’s post hoc test for comparing the differences between sham-operated mice and HI mice in the contralateral and ipsilateral hemispheres at each time point. n = 5 for sham group and n = 8 for HI groups. ns: not significant, *p < 0.05, **p < 0.01, ***p < 0.001. (PDF 138 kb) [file 12974_2018_1344_MOESM4_ESM.pdf]

Supplementary table 1. Statistical analysis of multiplex cytokine measurement in brain.

	IL1a	IL1b	IL2	IL5	IL6	IL9	IL10	IL12 (p40)	IL12 (p70)	IL13	IL17a	Eotaxin	G-CSF	GM-CSF	IFNg	KC	MCP-1	MIP-1a	MIP-1b	RANTES	TNFa
6H																					
Sham vs. Contra	ns	ns	ns	ns	ns	ns	ns	ns	ns	**	***	*	ns	ns	***	ns	ns	ns	ns	ns	ns
Sham vs. Ipsi	***	***	ns	ns	***	ns	***	ns	***	***	***	***	*	***	***	***	***	***	***	***	ns
Contra vs. Ipsi	***	***	ns	*	***	ns	***	ns	***	ns	ns	ns	*	***	*	***	***	***	***	ns	ns
1D																					
Sham vs. Contra	*	*	ns	ns	*	ns	*	ns	ns	***	***	***	ns	ns	***	ns	ns	ns	ns	**	ns
Sham vs. Ipsi	**	***	*	ns	***	ns	**	ns	ns	***	***	***	***	ns	***	ns	**	ns	ns	***	ns
Contra vs. Ipsi	ns	ns	ns	ns	ns	*	ns	ns	ns	ns	*	ns	*	ns	ns	ns	ns	ns	ns	ns	ns
3D																					
Sham vs. Contra	ns	ns	*	ns	ns	***	ns	ns	ns	*	**	**	ns	**	ns	ns	ns	ns	ns	ns	ns
Sham vs. Ipsi	***	***	***	*	*	ns	***	ns	ns	***	***	***	**	**	ns	ns	ns	*	ns	***	ns
Contra vs. Ipsi	**	**	*	**	ns	ns	**	ns	ns	*	*	ns	ns	ns	ns	ns	ns	ns	ns	**	ns
7D																					
Sham vs. Contra	ns	ns	ns	ns	ns	***	ns	***	***	ns	ns	*	ns	***	*	ns	ns	ns	ns	ns	ns
Sham vs. Ipsi	ns	ns	ns	ns	ns	ns	ns	ns	ns	ns	ns	ns	ns	ns	ns	ns	ns	ns	ns	ns	ns
Contra vs. Ipsi	ns	ns	ns	ns	ns	**	ns	***	***	ns	ns	*	ns	**	ns	ns	ns	ns	ns	ns	ns
14D																					
Sham vs. Contra	ns	ns	ns	*	*	ns	ns	ns	ns	ns	ns	ns	ns	ns	*	ns	ns	ns	ns	ns	**
Sham vs. Ipsi	***	ns	ns	ns	ns	ns	ns	***	**	ns	ns	**	ns	ns	ns	***	ns	ns	ns	***	ns
Contra vs. Ipsi	*	*	**	ns	ns	**	ns	***	*	ns	ns	**	ns	ns	ns	***	ns	ns	ns	***	ns

One-way ANOVA followed by Holm-Sidak's post-hoc test for comparing the differences between sham operated mice , HI mice contralateral and ipsilateral hemispheres at each time point. n= 5 for sham group and n= 8 for HI groups. ns: not significant, *p < 0.05, **p < 0.01, ***p < 0.001.
