# Supplementary material for: Peripheral myeloid cells contribute to brain injury in male neonatal mice
Source: J Neuroinflammation. 2018 Oct 30;15:301. doi: 10.1186/s12974-018-1344-9 (PMC6208095; doi:10.1186/s12974-018-1344-9)
Supplement: Supplementary file 5 — Table S2. Statistical analysis of multiplex cytokine measurement in plasma after HI. Student’s t test followed by Holm-Sidak’s post hoc test to correct for multiple comparisons between sham-operated mice and HI mice at each time point. n = 5 for sham group and n = 8 for HI groups. ns: not significant, *p < 0.05. (PDF 164 kb) [file 12974_2018_1344_MOESM5_ESM.pdf]

Supplementary table 2. Statistical analysis of multiplex cytokine measurement in plasma.

	IL1a	IL1b	IL2	IL3	IL4	IL5	IL6	IL10	IL12 (p40)	IL12 (p70)	IL13	IL17a	Eotaxin	G-CSF	GM-CSF	IFNg	KC	MCP-1	MIP-1a	MIP-1b	RANTES	TNFa
6H	ns	ns	ns	ns	ns	ns	ns	ns	ns	ns	ns	ns	ns	ns	ns	ns	ns	ns	ns	ns	ns	ns
1D	ns	ns	ns	ns	ns	ns	ns	ns	ns	ns	ns	ns	ns	*	ns	ns	ns	ns	ns	ns	ns	ns
3D	ns	ns	ns	ns	ns	ns	ns	ns	ns	ns	ns	ns	ns	ns	ns	ns	ns	ns	ns	ns	ns	ns
7D	ns	ns	ns	ns	ns	ns	ns	ns	ns	ns	ns	ns	ns	ns	ns	ns	ns	ns	ns	ns	ns	ns
14D	ns	*	ns	*	*	ns	ns	ns	ns	*	ns	ns	*	ns	ns	ns	ns	ns	ns	ns	ns	ns

Student *t*-test followed by Holm-Sidak's post-hoc test to correct for multiple comparisons between sham operated mice and HI mice at each time point. n= 5 for sham group and n= 8 for HI groups. ns: not significant, *p < 0.05
